# Occupational therapy and cooking: A scoping review and future directions

**DOI:** 10.1080/11038128.2023.2267081

**Published:** 2023-12-08

**Authors:** R. Hingst, D. C. Alvarado, L. Bardin, N. Farmer

**Affiliations:** a Rehabilitation Medicine Department, National Institutes of Health Clinical Center, Bethesda, Maryland, USA; b Translational Biobehavioral and Health Disparities Branch, Office of the Director, National Institutes of Health Clinical Center, Bethesda, Maryland, USA

**Keywords:** Culinary medicine, food skills, health promotion, meal preparation, multidisciplinary, population health, secondary prevention

## Abstract

**Background:**

Occupational therapy (OT) has historically used cooking as an intervention and assessment approach across settings. Current practices in OT and the emergence of the multidisciplinary field of culinary medicine highlight the relationship between cooking and health.

**Aims/Objectives:**

To map the current literature on OT and cooking and to identify key factors that may facilitate collaboration within culinary medicine.

**Materials and Methods:**

We conducted a scoping review using the Joanna Briggs Institute methodology to identify primary contexts and clinical settings. Publications were analysed using framework thematic analysis to identify OT themes and roles.

**Results:**

A total of 56 studies met the criteria for inclusion. The majority of studies (*n* = 29, 53%) represented home/community settings and brain injury was the largest clinical group (*n* = 15, 27%). Primary themes related to person (*n* = 47, 43%), occupation (*n* = 30 (28%), environment (*n* = 19, 17%), and psychosocial wellbeing (*n* = 13, 12%). The primary OT role identified was that of interventionist (*n* = 48, 86%).

**Conclusions/Significance:**

OT’s holistic practice places cooking within a larger context that can help identify and overcome the barriers to participation. Findings support multiple pathways in which OT can contribute to cooking initiatives for health promotion and potentially expand OT practice in population health.

## Introduction

Cooking is a nearly universal yet highly individualized occupation that crosses boundaries of practice areas, cultures, and contexts. Though occupational therapy (OT) uses terms such as *meal preparation, cookery, food skills,* and *food preparation,* in this paper, we use the general term *cooking* to encompass the process of preparing food for consumption. Cooking was one of the first occupations used in institutional settings even before the formalization of OT as a profession in the early twentieth century [1]. A search of early OT publications gives examples of therapeutic cooking in practice at a rehabilitation hospital kitchen [2], a women’s cottage at a long-term psychiatric institution [3], and a post-lobotomy programme at a veterans’ hospital [4].

Interest in cooking as a therapeutic modality continues in today’s healthcare field [5]. Occupational therapists routinely use cooking as an assessment, intervention, and targeted outcome in a variety of settings and with populations across the lifespan [5,6]. Occupational therapists have developed a variety of standardized assessments which use cooking to evaluate occupational performance or performance skills. The Assessment of Motor and Process Skills (AMPS), Rabideau Kitchen Evaluation-Revised (RKE-R), Kitchen Task Assessment (KTA), and Executive Function Performance Test (EFPT) use cooking in performance-based evaluation [6,7].

Cooking is a familiar task of daily living that involves integration of cognitive, physical, and psychosocial processes, which may contribute to a biopsychosocial impact on health. A systematic review of the psychosocial benefits of cooking concluded that its benefits can include general self-esteem, self-confidence, positive affect, and improved socialization [8]. However, the systemic review found that further qualitative and rigorous quantitative research is needed to identify and validate the mechanisms for psychosocial benefits of cooking [8]. Similarly, a recent systematic review found that though kitchen-related tasks, ranging from full meal preparation to single-step components, such as slicing, are commonly used by occupational therapists to improve clinical and functional outcomes for people with acquired brain injury, there currently is limited evidence supporting their use in this setting [5].

With regard to biological and physical health benefits, cooking interventions conducted in clinical and community settings are reported to improve dietary intake and related chronic disease risk factors, such as weight, blood pressure, and cholesterol levels [9]. The multidisciplinary field of culinary medicine has emerged to provide education related to meal planning and cooking in combination with counselling and repeated hands-on practice in order to promote health behaviour change [10]. Given the biopsychosocial implications from cooking, culinary medicine may provide a path for health promotion [11]. The culinary medicine team has generally included physicians, dietitians, public health specialists, and professional chefs. However, occupational therapists are not yet a prominent presence in the field. Given the well-established use of cooking within OT practice, there may be opportunities for occupational therapists to participate in health promotion through collaboration with culinary medicine.

In order to identify a role for OT in culinary medicine programmes, we decided to conduct a review of the literature to examine cooking in OT practice. We chose to undertake a scoping review as this method seeks to identify, map, report, and discuss concepts in the available literature on a topic of interest (e.g. OT and cooking), making this an appropriate method for this study [12]. While a systematic review was considered as a method, we determined that a scoping review could map the current literature and set a foundation for future systematic reviews to assess the quality of the evidence on focused topics of interest related to cooking in OT practice. This study had two aims: (1) to map the current literature on OT and cooking and (2) to use the findings to identify key factors that may facilitate collaboration within culinary medicine. We formulated the following research questions to guide the data collection and analysis:

- What is known from the literature about cooking in OT practice?
  - What are the contexts and clinical populations in which OT uses cooking?
  - What are the current roles of the occupational therapist?
  - What are the themes in OT literature regarding cooking?
  - What are the major findings regarding OT and cooking?
  - What are the major gaps in the current OT literature involving cooking?
- Based on the OT literature, what are the potential roles for occupational therapists in the field of culinary medicine?

## Methods

To answer the study questions, we conducted the scoping review using the Joanna Briggs Institute (JBI) methodology [13], which includes developing an a priori protocol detailing the research questions, objectives, inclusion/exclusion criteria, and methods. The final protocol was registered with Open Science Framework on 30 June 2022.

### Database search

We searched PubMed, PsycINFO, CINAHL Plus, and SCOPUS to locate published, peer-reviewed studies. Table 1 outlines the final search strategy. We also hand searched the reference lists of identified articles for additional relevant studies. We included all identified citations published up until 31 May 2022, collated and uploaded citations into Covidence software, and removed all duplicates [14].

### Inclusion and exclusion criteria

For this review, we defined cooking as the full process of preparing food for consumption, regardless of whether heat or appliances are used (e.g. no bake, salads, pre-cooked, etc.). A wide variation in terminology is used to describe cooking; therefore, we included terms such as cooking, meal preparation, food skills, etc. in this review. In order to focus specifically on cooking in OT in primary source, peer-reviewed studies, we established the following initial inclusion and exclusion criteria.

Preliminary inclusion criteria:

- Primary quantitative, qualitative, or mixed-methods study, including experimental, quasi-experimental, analytical observational, and descriptive observational study designs (including case series and individual case reports).
- Full text available in English or Spanish.
- Cooking engagement, evaluation, or intervention is central to the article.
- Must provide details about the cooking intervention, programme, or evaluation.
- Primary scope of the study is OT practice.
- Occupational therapist has substantial involvement in the design, intervention, evaluation, data collection, or analysis as indicated by authorship or the text of the article.

Preliminary exclusion criteria:

- Systematic reviews and meta-analyses.
- Articles that describe the intervention, practice, or programme without any data collection or analysis, such as protocols and opinion papers.

After an initial review of the full text and team discussion, we further refined the criteria to align with the research questions and removed from analysis articles that focused solely on the technology used in cooking without a description of the cooking process and articles that focused exclusively on assessment development, validation, or review. These are reported in Supplemental Table 1 for reference.

We also revised the criteria for defining cooking as central to provide greater clarity. If cooking was reported as part of a larger group of occupations (i.e. IADLs, food skills, therapeutic activities, etc.), articles needed to meet the following criteria:

- Provide independent discussion and/or analysis of cooking.
- Provide some level of detail/description of cooking activity/tasks.

### Study selection

Two independent reviewers (DA, RH) screened titles and abstracts for assessment within the Covidence software. After screening titles and abstracts, two independent reviewers (DA, RH) retrieved and assessed the full text of relevant studies in detail against the inclusion criteria. A third reviewer (LB) resolved disagreements between the reviewers at each stage of the selection process, including inclusion/exclusion and reasons for exclusion. Based on Covidence inter-rater reliability, the two reviewers (RH, DA) had a 93% proportion agreement and Cohen’s Kappa of 0.79. The majority of disagreements involved whether the article met the inclusion criteria of cooking being central. We reported the results in a Preferred Reporting Items for Systematic Reviews and Meta-analyses extension for scoping review (PRISMA-ScR) flow diagram [15].

### Data extraction

Two independent reviewers (DA, RH) extracted data points from included studies using the Data Extraction 2.0 tool in Covidence and consensus was reached. A third reviewer (LB) resolved disagreements. The data extracted included specific details about the context and clinical populations, role of cooking, roles of occupational therapists, major findings, and OT themes relevant to the research questions. We charted the data extracted from the studies in an Excel spreadsheet.

### Thematic analysis

To map common patterns in the OT literature, we analysed the themes identified during data extraction with a method based on the framework analysis approach [16]. This approach includes these main stages: (1) familiarization with the material (2) coding of initial themes (3) development of a framework for analysis based on the initial coding and (4) application of the framework to index the material [16]. First, we familiarized ourselves with the collected articles by reviewing the full texts and through data extraction. During the coding stage, we (DA, LB, RH) independently identified up to five themes related to OT in each study, answering the question, ‘What are the main constructs, concepts, or topics of this article?’ No a priori coding key was used for this process in order to include a variety of perspectives. Roles of occupational therapists and the contexts/clinical populations were not included as themes since data in these areas was extracted separately. After independent coding, we discussed the identified themes to develop the analytical framework. At this point, we considered whether relevant OT models and frameworks might serve to map the identified themes. The International Classification of Functioning, Disability, and Health (ICF), the Person-Environment-Occupation Model, the Person-Environment-Occupation-Performance Model, and the Model of Human Occupation were considered but did not clearly categorize all identified themes [17-20]. Therefore, we developed an analytical framework by categorizing the themes identified during coding, which incorporated language from the models listed above. See Supplemental Figure 1 for the framework developed for analysis. Next, to provide a concise overview of the literature, two researchers (DA, RH) independently indexed the included studies using the developed framework by assigning no more than two primary themes to each study. The two researchers (DA, RH) reached a consensus on two primary themes per article through discussion and then a third researcher (LB) validated the assignments. We used the same approach to develop a framework to analyse occupational therapist roles represented in the articles, with each article having up to two primary roles. The initial OT role codes were assigned by researchers independently by asking, ‘What is the contribution of or services provided by the occupational therapist in this article?’ See Supplemental Figure 2 for the analytical framework developed for indexing OT roles.

## Results

**Figure 1.**
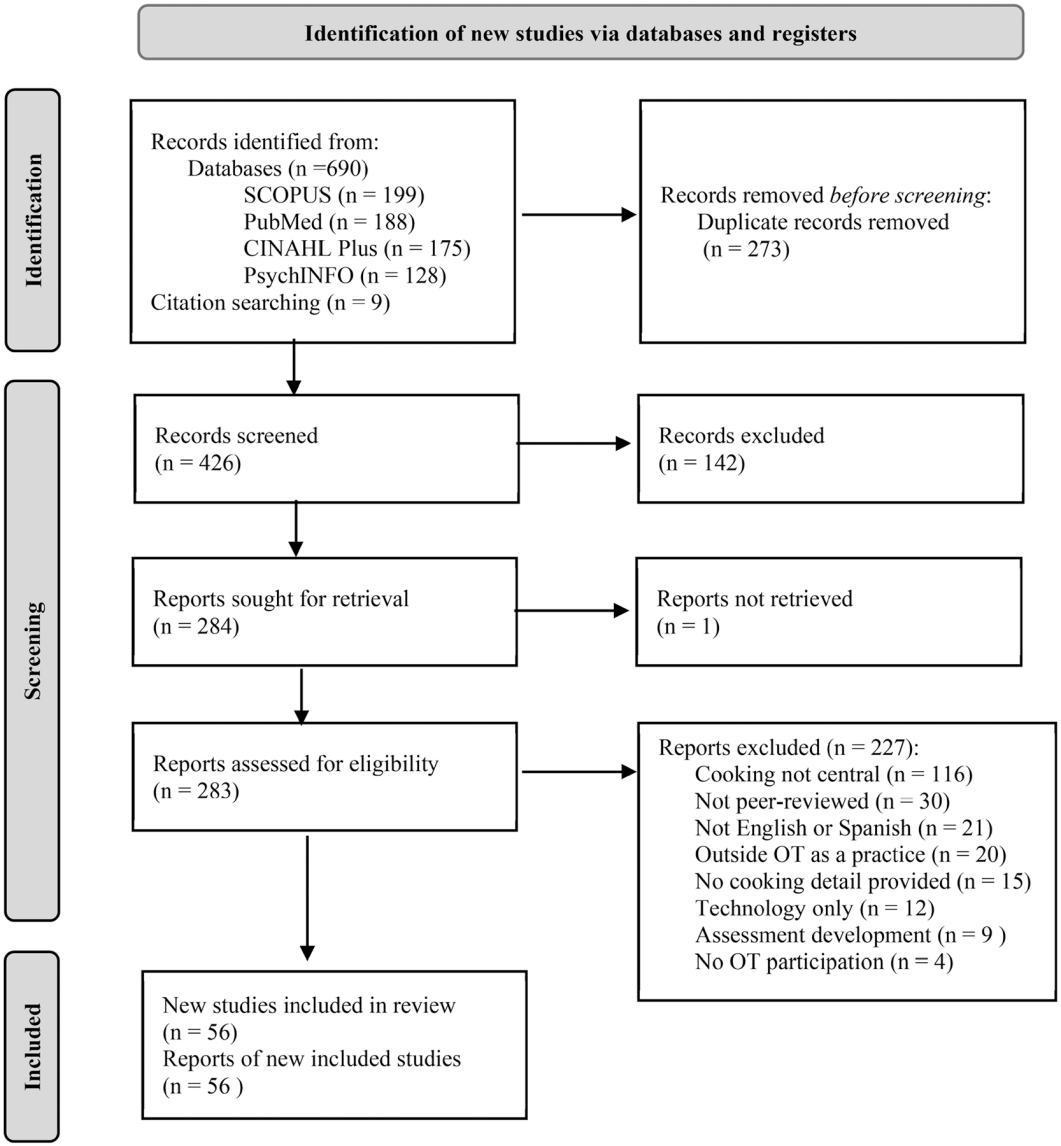
PRISMA diagram.

A total of 690 studies were gathered from searches of the following databases: PubMed (*n* = 188), PsycINFO (*n* = 128), CINAHL Plus (*n* = 175), and SCOPUS (*n* = 199). An additional nine (*n* = 9) articles were identified by reviewing the reference lists of identified studies. After the removal of duplicates, 426 studies remained for the title and abstract screening. See PRISMA diagram (Figure 1) for details.

Title and abstract screening were completed in the Covidence software, resulting in 142 excluded studies and 284 remaining for full-text review. We retrieved a total of 283 full-text articles for review, and one article was not located through library services. A total of 227 studies were excluded after full-text review for the following reasons: cooking not central (*n* = 116); not peer-reviewed (*n* = 30); not available in English or Spanish (*n* = 21); outside OT as a practice (*n* = 20); no cooking detail provided (*n* = 15); technology only (*n* = 13); assessment development/review (*n* = 8); and no occupational therapist participation (*n* = 4).

### Description of studies

**Figure 2.**
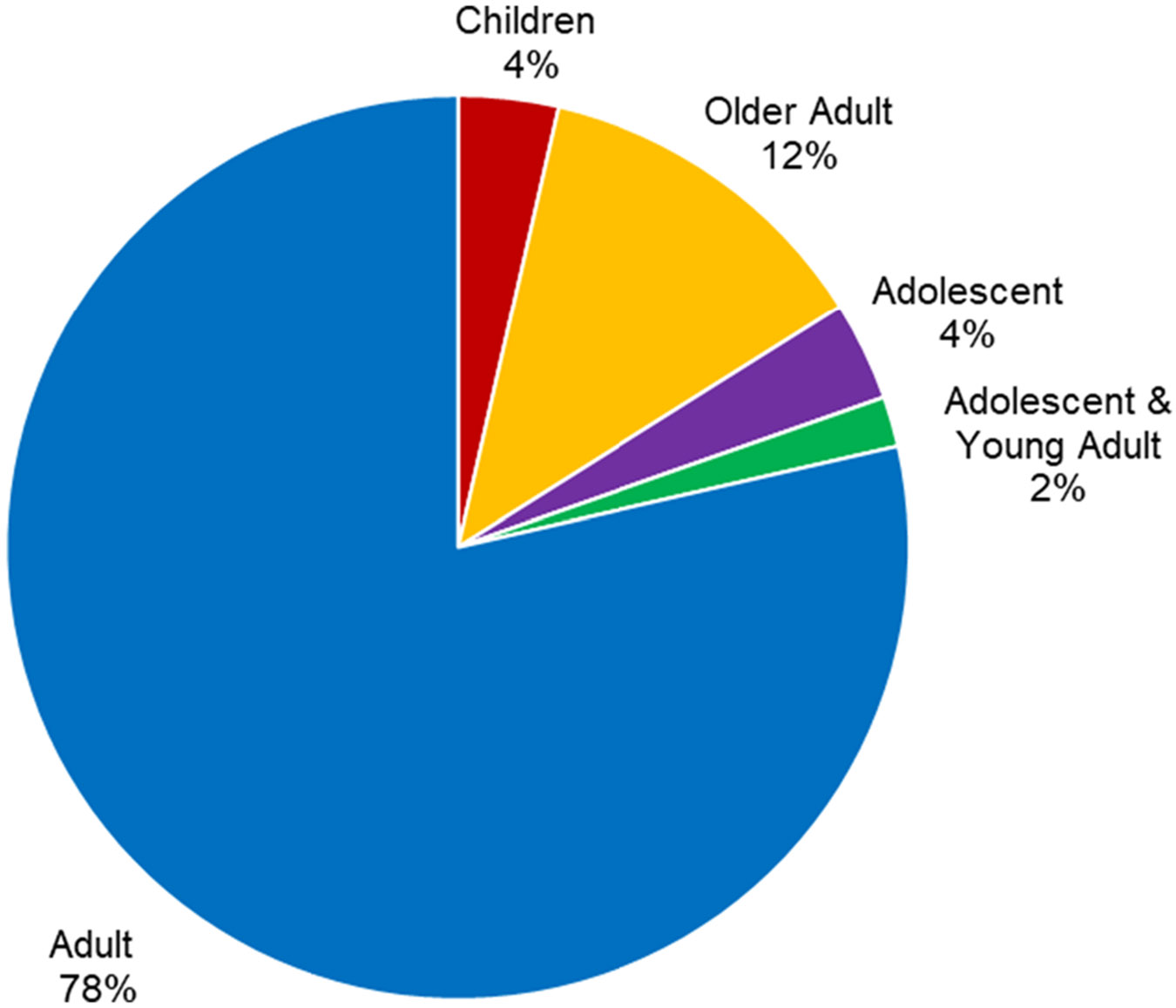
Descriptive statistics by age group^a^. ^a^Age group as defined/described by study authors

**Figure 3.**
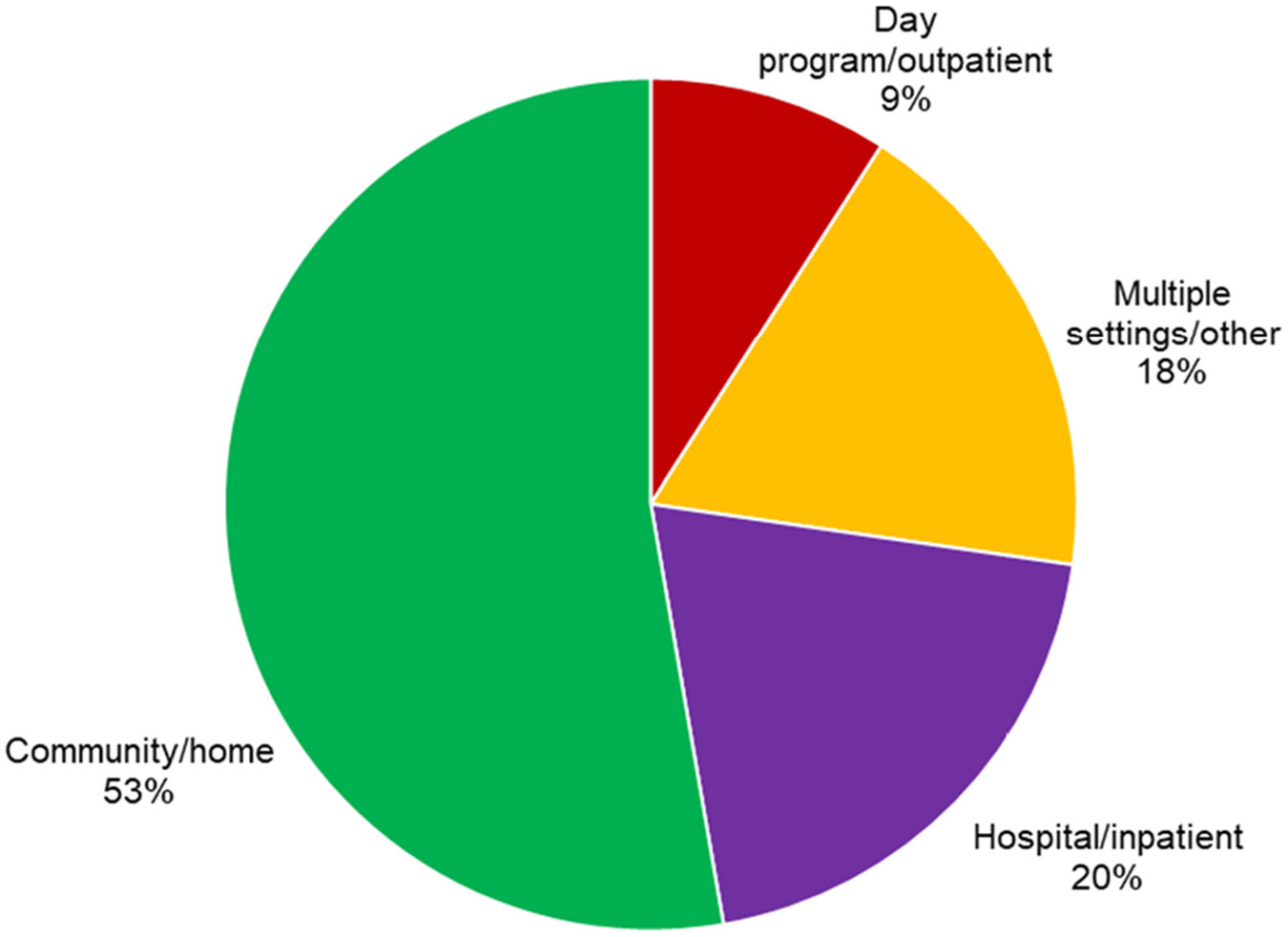
Descriptive statistics by practice settings.

**Figure 4.**
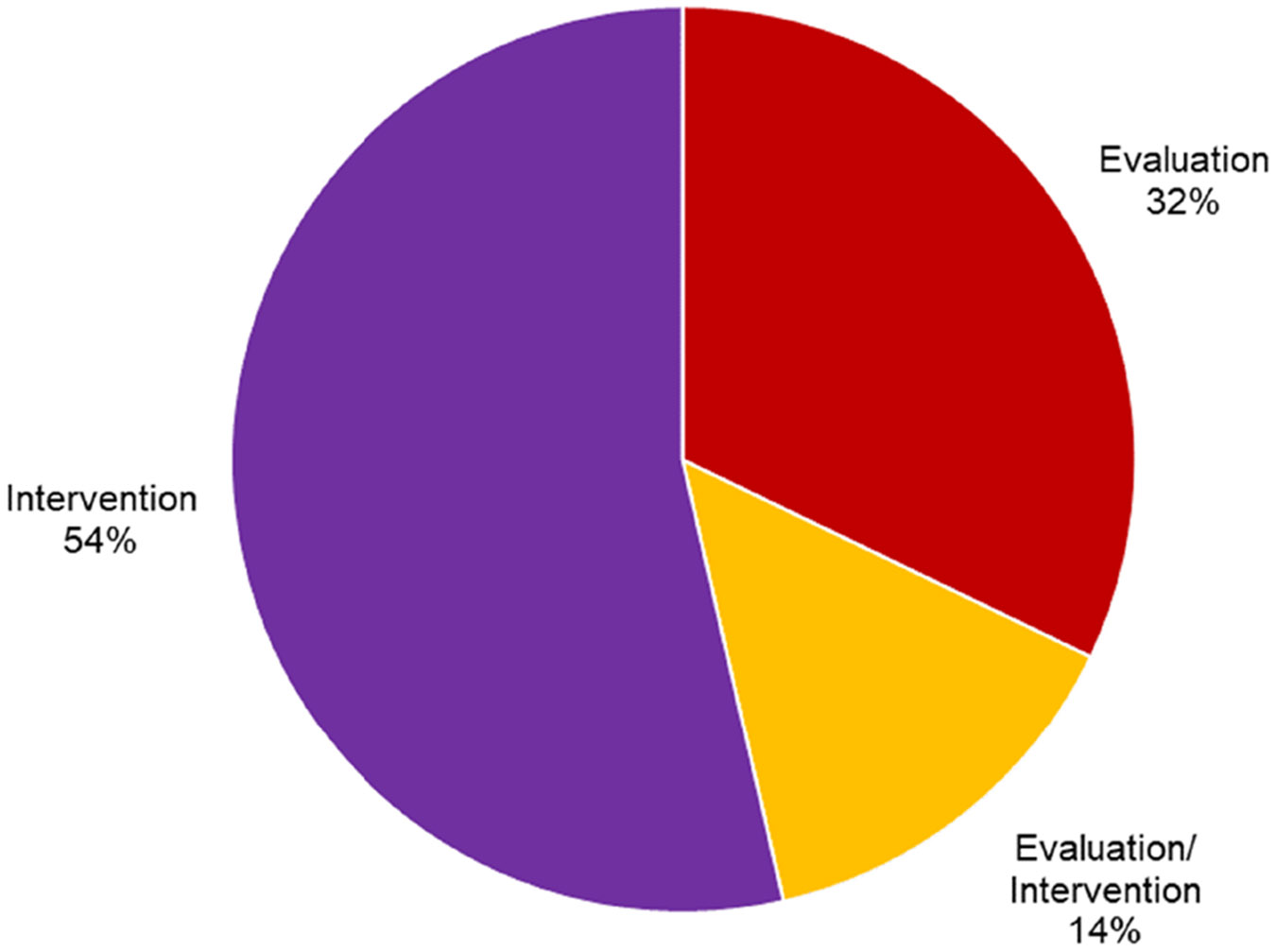
Descriptive statistics by occupational therapy process.

A total of 56 articles met the eligibility criteria for inclusion. Supplemental Table 2 lists these by their first author and presents the population, context, role of cooking, and major findings. The studies represented 18 clinical groups across the lifespan (Table 2, Figure 2). Four settings of practice were identified, with community/home settings accounting for 53% (*n* = 29) of the study settings (Figure 3). The majority of studies (54%) analysed cooking as an intervention, while 32% reported cooking as an evaluation and 14% used cooking as a combination of evaluation and intervention (Figure 4). The studies included were conducted across eleven countries (Table 3). After thematic analysis, eleven (*n* = 11) unique occupational therapist roles were reported in the 56 studies, with 23 studies having two primary roles and 33 having one. These roles and their corresponding studies are listed in Table 4. A total of 31 unique primary OT themes (Table 5) were identified based on 56 studies, with 53 studies having two primary themes and three having one.

### Role of occupational therapist

The most prominent role identified was that of interventionist (*n* = 48, 86%), with occupational therapists using a variety of approaches to promote cooking performance and participation. The primary roles for intervention included skills training (*n* = 24, 30%) and group facilitation (*n* = 17, 22%). Occupational therapists were also acknowledged for their role in evaluation, most frequently through performance-based assessment of cooking (*n* = 18, 23%).

### Theme: person

Forty-two (*n* = 42) papers addressed person-level themes related to cooking, accounting for 43% of all identified themes. The relationship between cognition and cooking was significant in the literature, with 27 assigned themes focusing on aspects of cognition. In addition to the theme of general cognition (*n* = 7), a specialized focus was given to executive functioning (*n* = 8) and metacognition/self-awareness (*n* = 5).

Personal competencies (*n* = 7) are another person-level factor noted to play a role in cooking performance. This encompasses studies that considered a broader profile of body functions and performance skills in subjects. Several studies examined the association between a range of personal competencies and cooking performance in diagnostic groups such as burns [21], stroke [55], mental illness [22], physical disabilities [51], visual impairments [23], and age-related impairments [56,57]. Other studies noted that functional performance was dependent on a participant’s level of personal competencies in relation to activity demands [73,75]. Two additional studies focused specifically on motor performance during cooking tasks [24,55].

Activity-related knowledge and skills (*n* = 10) were also recognized for their impact on cooking performance and as outcomes of cooking interventions. Several interventions emphasized specific skill acquisition, focusing on food skills [25,26], preparing specific meals [27], maximizing food resources [28], and developing social skills [27,45].

### Theme: occupation

Thirty (*n* = 30) articles examined cooking as an occupation, with focus on task-level factors and supports to promote participation. Occupational therapists frequently promoted performance through the structure and approach (*n* = 16) of cooking, such as altering the type of feedback [29,30], promoting metacognitive and cognitive reflection [31,32,46,58], providing opportunities for hands-on learning [33-36], structuring group interventions [37,38], and suggesting modification for tasks [75].

The value and meaning (*n* = 8) attached to cooking were described as motivators for participation and outcomes of OT interventions. The value and importance of cooking [39,47,48,59], the meaning of accessibility and choice in the kitchen [52,57], and a sense of purpose [40,49] were associated with cooking participation. Cooking was also emphasized as an occupation connected to general self-maintenance (*n* = 2) [25,41] and community living (*n* = 3) [60-62] as well as health management (*n* = 1) [50].

### Theme: environment

Eighteen (*n* = 18) studies highlighted the influence of context and environment on cooking, inclusive of physical and social factors in the cooking space. Directly comparing performance in the clinic versus the home emerged as a major theme (*n* = 5). The second major theme was the implementation of technology to support cooking performance (*n* = 5). Wider contextual factors also played a role in cooking interventions and participation, such as institutional/programmatic factors (*n* = 2) [22,49], policy standards (*n* = 1) [74], and socioeconomic situations (*n* = 1) [28].

### Theme: psychosocial wellbeing

Thirteen (*n* = 13) studies highlighted the psychosocial benefits derived from cooking engagement. This includes general improvements in quality of life and wellbeing (*n* = 6) associated with cooking participation. More specially, studies identified positive affective responses [47,48], increased motivation for cooking [37], opportunities for reminiscing and related benefits [46], and reduction in functional concerns and anxiety [21,50]. In three studies (*n* = 3), participants elaborated on the value of cooking as a group and its contribution to mental health recovery [37,39,42]. Similarly, improved self-efficacy and empowerment were noted as a result of cooking through skill development [49], experiences facilitated by the occupational therapist during cooking [40], and maintenance of cooking participation while ageing [73].

## Discussion

To summarize, we can see that the OT literature on cooking spans clinical groups and reflects a broad range of themes related to person, environment, occupation, and psychosocial wellbeing. The results also illustrate occupational therapists working in a variety of settings in multiple roles to promote participation in cooking through skills training, adaption of tasks, performance-based assessment, and consideration of the environment. These findings can guide us in addressing the second study aim: to identify factors that may facilitate collaboration within culinary medicine. The broad knowledge base and specialized skillset highlighted by the review has the potential to be applied to culinary medicine, a multidisciplinary field rooted in health promotion. In this discussion, health promotion is defined in the words of the Ottawa Charter for Health Promotion [77] as ‘the process of enabling people to increase control over, and to improve, their health.’

### Health promotion as a context for collaboration

Future directions for OT and cooking include moving beyond clinical practice into occupation-based health promotion. Leaders in the OT field, such as therapist, scientist, and theorist Ann Wilcock, have long advocated for the use of occupation to promote health and wellbeing in populations [78]. Examining the themes reported in Table 5 helps link cooking in clinical OT practice with health promotion and to identify the approaches that occupational therapists can bring to culinary medicine programmes. By using health promotion as a context for collaboration, there is potential for culinary medicine and OT to advance a shared goal of developing interventions and expanding the evidence base for using cooking to enhance health and wellbeing.

Health promotion often focuses on individuals or populations initiating and sustaining a change in health behaviour. In culinary medicine, behaviour change interventions concentrate on starting or changing cooking behaviour at home because there is strong evidence in the literature that cooking at home improves diet quality and related health outcomes [79]. While culinary medicine describes cooking as health behaviour, the broad scope of themes in the review findings reinforce that OT understands cooking as a complex, multidimensional process with layers of meaning [80]. These are complimentary but distinct views of cooking that bring different knowledge bases and intervention approaches to the table.

Tucker et al. [81] identified client-centred practice and a holistic approach as two areas that provide synergistic opportunities for OT and health promotion. The range of themes across person, occupation, environment, and psychosocial wellbeing in the review results reflects the holistic nature of OT practice. Person-level themes are the most common primary themes that emerged from the review, which highlight occupational therapists’ client-centredness and their expertise in person-level factors that underpin cooking performance and skill acquisition. While knowledge and skills are included as components in behaviour change models used in health promotion, they are not described in detail. However, occupational therapists’ work with clinical populations allows for a comprehensive yet nuanced understanding of how physical, cognitive, mental, and social competencies affect performance of daily activities, which can be applied to promote health.

Value and meaning also emerged as a significant theme in OT cooking literature. This is not surprising because OT recognizes the value and meaning of occupation as essential for client-centred practice. In fact, OT scholar Charlotte Royeen [82] defined *occupation* simply and eloquently as ‘doing with meaning.’ Occupational therapists understand how the value and meaning of cooking relate to motivation, a key construct in models of health behaviour change [83,84]. A focus on client-centred practice places occupational therapists in an excellent position to explore and leverage a person’s motivation to facilitate sustained behaviour change [81].

Using a holistic approach, OT sees the cooking process as part of a larger and more complex pattern of daily activities rather than a discrete health behaviour. Recognizing temporal patterns and connections between occupations could be a key factor in supporting the integration of newly acquired skills from culinary medicine interventions into daily routines. Occupational therapists understand the interconnections between cooking and related occupations such as shopping, meal planning, community mobility, financial management, leisure, social participation, eating, and caregiving. While this review focused on literature where cooking was central, the findings nevertheless show cooking was entwined with themes such as community living, self-maintenance, health management, food skills, and social participation. There are other examples in the OT literature examining cooking in conjunction with related occupations [85-93].

### Multiple perspectives on context and the environment

OT has long considered the environment as central to the understanding of occupational performance, and environment-level themes are well-represented in the review results. Similarly, culinary medicine is interested in how environmental factors, such as availability of ingredients in an individual’s food environment, facilitate or limit engagement in cooking to promote health [94]. Both of these views align with the Ottawa Charter for Health Promotion, which identifies creating supportive environments as one of its five action areas. While OT is certainly not alone in its appreciation of the environment’s influence on health, the review results demonstrate how OT explores and addresses this relationship on both micro and macro levels. Examples from the review results show OT addressing the specific task environment to promote participation in cooking, including assessing and modifying how the physical space is set up, directly comparing performance in different environments, and employing assistive devices and technology. There is further evidence in the OT literature of using specific technology and modification in the kitchen environment to overcome person-level limitations and of assessing safety in the kitchen setting [95-102]. Additionally, the findings also highlight how the household and social environment, such as the challenges of a shared living space, influences the acquisition of skills, motivation, and engagement in cooking [26,52,75].

In terms of wider context, OT is concerned with macro factors like the influence of public policy, which links to the Ottawa Charter’s action area of building healthy public policy. The review results include examples of how housing accessibility policies, public funding for environmental modification, and the occupational deprivation of poverty affect engagement in cooking [28,52,74]. The wider OT literature includes further evidence of OT’s concern about the environment’s impact on food provisioning, such as how access to shops, transportation, and concerns about community safety affect participation in food acquisition and preparation [103-106]. This broad yet practical understanding of the cooking environment can support the acquisition of cooking skills in culinary medicine interventions and the successful translation of these skills to home settings.

### An occupation-based health promotion intervention

Scaffa et al. [107] urged occupational therapists to emphasize occupation as an essential element of health promotion strategies. Likewise, culinary medicine programmes frequently use hands-on cooking classes to teach the skills necessary for preparing nutritious meals that will prevent, manage, and reverse chronic diseases [11]. This approach links to the Ottawa Charter’s action area of developing personal skills. As experts in occupation-based intervention, occupational therapists have the potential to make a significant contribution to personal skill development through supporting culinary medicine programme design and implementation. The review results also affirm occupational therapists’ role as expert interventionists, particularly in skills training and group facilitation. For example, articles describe evaluating a range of techniques to facilitate skill acquisition, including specific strategies targeting metacognition, information processing, and executive function. Occupational therapists also incorporate psychosocial skills training in cooking, such as addressing social skills [27,38,39] and facilitating the use of coping skills within the context of cooking [21,37]. Similarly, OT’s long tradition of group interventions is reflected with examples across practice areas and clinical populations. Since many culinary medicine programmes focus on teaching skills in group settings, occupational therapists are a natural fit for leading or co-facilitating interventions.

Additionally, occupational therapists can provide an in-depth perspective of the cooking process by employing OT models of occupational performance and systems of activity/task analysis to facilitate personal skill development. The process of breaking tasks down and identifying the underlying skills necessary for the cooking process is the first step in modifying interventions to meet specific needs. In health promotion, this is congruent with a process referred to as *tailoring* and is of particular interest when working with underserved communities [108]. In culinary medicine practice, tailoring focuses on matching dietary assessment, recommendations, and interventions to personal goals and readiness for change [109]. Occupational therapists can collaborate with the culinary medicine team to create and use tailored support with participants, instructors, and programme developers [110-112].

The review also identified assessment of cooking performance as a significant role for occupational therapists. As of yet, culinary medicine research has not focused on cooking skills as a primary outcome and has generally relied on self-reported measures to quantify cooking performance [113]. Occupational therapists’ expertise in evaluating underlying skills and using standardized performance-based assessments is valuable in identifying needs of target populations and in researching outcomes of hands-on culinary medicine interventions [111,113].

### An opportunity for population health practice

As detailed in Table 2, OT has used cooking with a range of clinical groups, from brain injury to mental illness to visual impairments. Health promotion provides occupational therapists opportunities to apply specialized skills used to address physical, psychosocial, or cognitive impairments in clinical practice to a wider population. In the context of health promotion, OT and culinary medicine share a population of interest: the at-risk population. This is the target population for secondary prevention interventions, including culinary medicine interventions, which seek to limit and prevent further decrease in health status in those at risk.

Serving at-risk groups aligns with the fourth action area from the Ottawa Charter: reorienting health systems to balance treatment with more preventative approaches. While culinary medicine has typically used biomarkers such as blood pressure and cholesterol levels to define at-risk populations, as a rehabilitation profession, OT is concerned with measures of function and participation. Both are relevant when considering target populations for health promotion interventions that embrace the WHO’s holistic definition of health as ‘complete physical, mental and social well-being and not merely the absence of disease or infirmity [114].’

WHO’s ICF describes health and functioning on a continuum, with people experiencing varying degrees of impairments and limitations depending on body structures and functions and environmental factors [18]. Though approximately 15% of the world’s population can be considered to have a moderate to severe disability–those typically seen in traditional OT practice–an even larger number of people live with a milder degree of physical, psychosocial, or cognitive impairment that limits functioning [115]. According to the World Report on Disability [115], 16% of people worldwide report mild difficulty moving around, 26% report mild bodily aches and pains, 20% report mild difficulty with concentrating and remembering, and 22% report mild feelings of depression. These types of challenges describe this shared population of interest because they likely result in difficulty accessing, engaging in, and benefiting from health promotion interventions, such as hands-on cooking, that have the potential to prevent further limitations or disability.

The final action area from the Ottawa Charter is strengthening community action, which harmonizes with OT’s occupational justice framework [116,117]. Though this area, described as drawing on community resources for self-help and social support, is less reflected in the review results, it appears to be an important potential area for collaboration with culinary medicine. Looking through the lens of occupational justice and social justice, there are other at-risk populations defined by their social or economic vulnerability for whom cooking interventions may serve as a catalyst for community involvement and self-help. Indeed, the theme of psychosocial wellbeing which emerged from the review may be especially relevant within these communities and this is an area that needs further exploration. These additional at-risk populations include those experiencing chronic stress and limited resources, such as people who are displaced, those in conflict settings, and those exposed to other violence, systematic discrimination, and poverty [118-120]. Cooking is an intervention that encompasses both biomedical-based health outcomes, such as nutrition status, and outcomes focusing on psychosocial health and quality of life that are of great concern in these groups. There is already evidence in the literature that occupational therapists have explored cooking and food security with vulnerable groups [28,87,121-123] and partnering with culinary medicine can provide further opportunities to promote occupational justice.

### Gaps in the literature

Though the OT literature covers multiple themes, contexts, and populations, there is limited evidence across practice areas. Neurorehabilitation and mental health are the most frequently represented clinical areas, but many other diagnostic groups familiar to OT practice are minimally or not represented. Although the review was limited to English and Spanish language publications, we found few articles from authors or settings in low- and middle-income countries. While this review did not assess methodology, it is apparent from the overview that there are limited studies that used high-quality methodology and experimental studies have not been replicated in multiple settings or trials.

### Methodological considerations and limitations

An initial limitation of this review is that it was restricted to English and Spanish language publications to avoid errors in interpretation; this approach likely excluded relevant articles from diverse settings. Second, to focus on cooking in clinical OT practice, the review excluded occupational science articles that could provide further insight into cooking as an occupation and how it is experienced in non-clinical settings. Third, though we made a concerted effort to clearly define inclusion criteria and used multiple reviewers for study selection, there was a certain degree of subjectivity in the interpretation of these criteria. Finally, as a scoping review seeking to provide a broad overview of the topic, we did not report on the methodology or assess the quality of the evidence gathered in the review. Similarly, to provide a summary of the literature, we reported only the most prominent themes from each study. These limitations and biases may have resulted in a narrower pool of studies included in the review and a more focused but a less nuanced and diverse view of cooking in OT practice. However, we reached a consensus at all stages, and we do not believe this affected the general results and overall conclusions of the study. On a positive note, this is the first scoping review on this topic, and its methodological strengths include the use of multiple, independent reviewers at all stages.

### Implications for the future

The results have two major implications for OT. First, further descriptive research related to cooking is needed for multiple populations in all practice areas, which can highlight OT’s value in a multidisciplinary field like culinary medicine. This research would provide a foundation for the development of both clinical and population health interventions that can be evaluated through rigorous research methods to strengthen the evidence base. Due to the complexity of cooking as an occupation, mixed methods research has the potential to provide rich, contextualized, and generalizable data in both descriptive and experimental designs. Secondly, this scoping review affirms that OT has a broad knowledge base and specialized skillset with the potential to contribute to culinary medicine in multiple roles. Though culinary medicine programmes and funding sources vary by region and country, OT can be proactive in promoting the profession’s expertise and seeking roles in the field. Based on the review findings and how they relate to key components of health promotion and culinary medicine programmes, we propose the following potential roles:

- Partners in programme design and development, especially when considering the needs of at-risk populations.
- Consultants for increasing access and overcoming barriers to participation in culinary medicine programmes, particularly with vulnerable groups.
- Facilitators for hands-on programmes, specifically adapting tasks and the environment to match participant needs and using client-centred approaches to build motivation to establish new health behaviours.
- Collaborators in research on culinary medicine, particularly bringing expertise in ‘doing’ and in measurement of holistic outcomes
- Innovators in the use of cooking spaces in facilities, proactively considering how to use cooking spaces to serve broader populations, involving other disciplines, and creatively using available resources for cooking interventions.
- Advocates and allies in promoting policies that support food security, improve the food environment, and address barriers to cooking participation together with vulnerable groups and communities.

## Conclusion

In summary, this review highlights the specialized knowledge and skills occupational therapists can bring to a multidisciplinary team seeking to promote health through cooking, including understanding the occupational context, utilizing specific strategies for skill acquisition based on a person-centred approach, and making adaptations to overcome barriers and limitations. The OT literature reflects a broad range of themes related to person, environment, occupation, and psychosocial wellbeing, which support multiple potential roles within culinary medicine programmes. However, there are currently a number of gaps in the literature and a need to assess the quality of the methodology and evidence in this area. Future directions for OT include both building the field’s evidence base and partnering with culinary medicine to expand practice in population health.

## Supplementary Material

Supplemental Fig 1

Supplemental Fig 2

Supplemental References

Supplemental Table 1

Supplemental Table 2

## Figures and Tables

**Table 1.** Search strategy.

Database	*n*=	Search Strategy
PubMed	188	(‘occupational therapy’ OR ‘occupational therapist’ OR ‘occupational therapists’ OR ‘OT’) AND (‘cooking’ OR ‘meal preparation’ OR ‘baking’ OR ‘kitchen’ OR ‘food skills’)
PsychINFO	128	(Any Field: ‘occupational therapy’ OR Any Field: ‘occupational therapist’ OR Any Field: ‘occupational therapists’ OR Any Field: ‘OT’) AND (Any Field: ‘cooking’ OR Any Field: ‘meal preparation’ OR Any Field: ‘baking’ OR Any Field: ‘kitchen’ OR Any Field: ‘food skills’) AND Peer-Reviewed Journals only
CINAHL Plus	175	(‘occupational therapy’ OR ‘occupational therapist’ OR ‘occupational therapists’ OR ‘OT’) AND (‘cooking’ OR ‘meal preparation’ OR ‘baking’ OR ‘kitchen’ OR ‘food skills’) Limiters – Peer Reviewed Expanders – Apply equivalent subjects Search modes – Boolean/Phrase
SCOPUS	199	TITLE-ABS-KEY ((‘occupational therapy’ OR ‘occupational therapist’ OR ‘occupational therapists’ OR ‘OT’) AND (‘cooking’ OR ‘meal preparation’ OR ‘baking’ OR ‘kitchen’ OR ‘food skills’)) AND (LIMIT-TO (SRcTYPE , ‘j’))

**Table 2.** Descriptive statistics of articles by clinical group (*n* = 56).

Clinical Group	*n* (%)
Brain injury	15 (27)
Schizophrenia	8 (14)
Mental illness, not specified	7 (13)
Ageing	6 (11)
Executive function deficit	3 (5)
Healthy	3 (5)
Stroke	3 (5)
Autism Spectrum Disorder	1 (2)
Burns	1 (2)
Cancer	1 (2)
Cognitive disabilities	1 (2)
Dementia	1 (2)
Eating disorders	1 (2)
Physical & psychosocial disabilities	1 (2)
Physical disability	1 (2)
Poverty	1 (2)
Sickle cell disease	1 (2)
Visual impairments	1 (2)

**Table 3.** Descriptive statistics of articles by country (*n* = 56).

Countries	*n* (%)
USA	18 (32)
Canada	11 (20)
Australia	8 (14)
UK	7 (13)
France	3 (5)
Israel	3 (5)
Japan	2 (4)
Denmark	1 (2)
Italy	1 (2)
Netherlands	1 (2)
Sweden	1 (2)

**Table 4.** OT roles (*n* = 76)^a^.

OT Role	*n* (%)	Articles
Interventionist	48 (86)	
Skills training	24 (30)	[21-44]
Group facilitation	17 (22)	[21,22,25,27,28,35,37-39,42,44-50]
Environmental modification	4 (5)	[23,51-53]
Therapeutic use of self	2 (3)	[40,45]
Activity modification	1 (1)	[54]
Evaluator	29 (52)	
Performance-based assessment	18 (23)	[55-72]
Activity analysis	7 (9)	[52,58,60-63,73]
Environmental assessment	2 (3)	[73,74]
Needs assessment	2 (3)	[41,75]
Other roles	2 (1)	
Expert opinion	1 (1)	[76]
Multidisciplinary team process	1 (1)	[50]

a 56 studies with total 76 identified roles (23 studies having two primary roles and 33 having one primary role).

**Table 5.** OT themes (*n* = 106)^a^.

Theme	*n* (%)	Articles
Person	47 (43)	
Cognition (General)	7	[32,53-55,61,69,76]
Executive functioning	8	[38,44,58,64-67,71]
Metacognition/self-awareness	5	[29-31,34,66]
Information processing	3	[60,62,63]
Constructional skills	2	[43,70]
Memory	2	[33,36]
Personal competencies (General)	7	[22,23,51,56,57,73,75]
Knowledge/skills (General)	4	[26,35,41,63]
Food skills	4	[25,27,28,70]
Social skills	2	[27,45]
Motor performance	2	[24,55]
General health	1	[72]
Occupation	30 (28)	
Structure/approach	16	[21,24,29-38,43,46,58,75]
Value and meaning	8	[39,40,47-49,52,57,59]
Community living	3	[60-62]
Self-maintenance (General)	2	[25,41]
Health management	1	[50]
Environment	19 (17)	
Clinic vs home	5	[26,56,68,71,72]
Technology	5	[23,51,53,54,76]
Institutional/programmatic	2	[22,42]
Naturalistic context	2	[65,69]
Physical (home)	2	[52,74]
Clinic	1	[59]
Policy/political	1	[74]
Socioeconomic	1	[28]
Psychosocial wellbeing	13 (12)	
General/quality of life	6	[21,46-48,50,64]
Mental health recovery	3	[37,39,42]
Self-efficacy/empowerment	3	[40,49,73]
Social participation	1	[45]

a 56 studies with 106 total identified themes (53 studies having two primary themes and 3 having one primary theme).
