# Supplementary material for: Occupational therapy and cooking: A scoping review and future directions
Source: Scand J Occup Ther. Author manuscript; Available in PMC 2024 Dec 12. (PMC11635739; doi:10.1080/11038128.2023.2267081)
Supplement: Supplemental Fig 1 [file NIHMS2038246-supplement-Supplemental_Fig_1.pdf]

Supplemental Figure 1. Framework analysis of OT themes

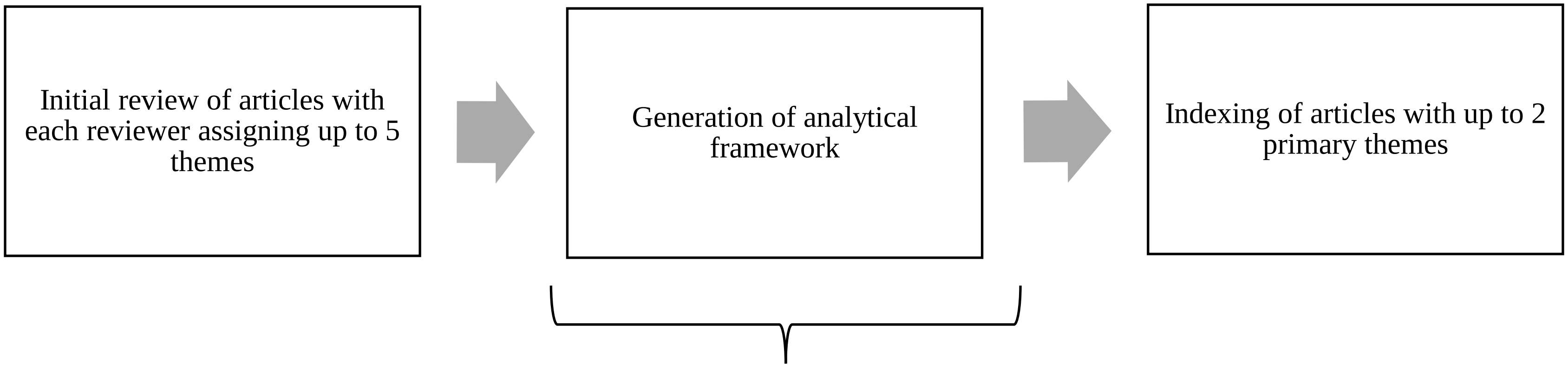

**Person**	**Environment**	**Occupation**	**Psychosocial wellbeing**
Personal competencies<br>Motor performance<br>Cognition<br>Executive function<br>Memory<br>Metacognition/self-awareness<br>Skills/Knowledge<br>Social skills<br>Food skills<br>Psychological[a]<br>General health	Socio-economic<br>Institutional/programmatic<br>Political/policy<br>Physical[a]<br>Household<br>Technology<br>Clinic<br>Clinic v. Home<br>Social[a]<br>Naturalistic context/ecological	Self-maintenance<br>Health management/diet quality<br>Money management[a]<br>Community living<br>Task modification[b]<br>Structure/approach<br>Value & meaning	General/Quality of life<br>Mental health recovery<br>Peer support/social participation<br>Self-efficacy/empowerment

[a] Theme not assigned as top 2 primary themes during indexing
[b] Theme was subsumed under *Structure/approach* during indexing
