# Supplementary material for: Occupational therapy and cooking: A scoping review and future directions
Source: Scand J Occup Ther. Author manuscript; Available in PMC 2024 Dec 12. (PMC11635739; doi:10.1080/11038128.2023.2267081)
Supplement: Supplemental Fig 2 [file NIHMS2038246-supplement-Supplemental_Fig_2.pdf]

Supplemental Figure 2. Framework analysis of OT roles

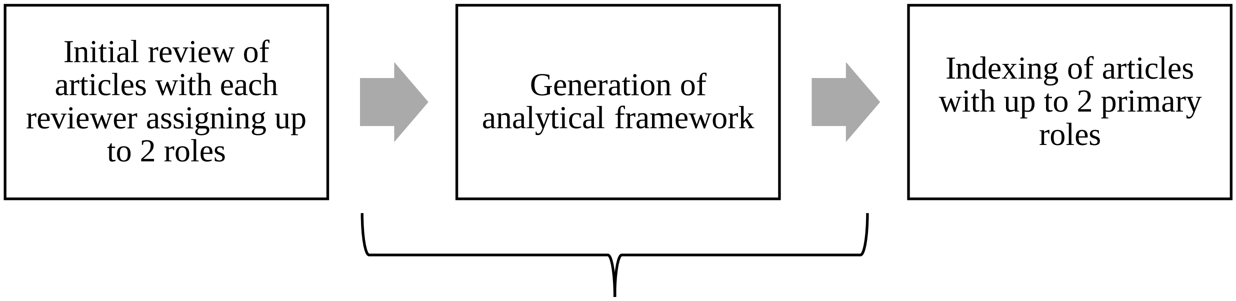

Assessment Roles	Intervention Roles
Activity analysis	Activity modification
Environmental assessment	Environmental modification
Needs assessment	Group facilitation
Performance-based assessment	Expert opinion[b]
	Advocacy[a]
	Study design and[a] implementation
	Multidisciplinary team process[b]
	Skills training
	Skills training
	Caregiver training[a]
	Intervention supervision[a]
	Therapeutic use of self

[a] Role not assigned as primary role during indexing

[b] Role was moved from category of "interventionist" to "other" in reporting of results for clarity
