# Supplementary material for: Occupational therapy and cooking: A scoping review and future directions
Source: Scand J Occup Ther. Author manuscript; Available in PMC 2024 Dec 12. (PMC11635739; doi:10.1080/11038128.2023.2267081)
Supplement: Supplemental References [file NIHMS2038246-supplement-Supplemental_References.pdf]

References

1. Catalan JM, Blanco A, Bertomeu-Motos A, et al. A Modular Mobile Robotic Platform to Assist People with Different Degrees of Disability. Appl Sci. 2021;11(15):7130.
2. Ellington A, Adams R, White M, et al. Behavioral Intention to Use a Virtual Instrumental Activities of Daily Living System Among People With Stroke. Am J Occup Ther. 2015;69(3):p1-8.
3. Johansson K, Lundberg S, Borell L. "The Cognitive Kitchen" - Key principles and suggestions for design that includes older adults with cognitive impairments as kitchen users. Technol Disabil. 2011;23(1):29-40.
4. Kirshner S, Weiss PL, Tirosh E. Meal-Maker: A virtual meal preparation environment for children with cerebral palsy. Eur J Spec Needs Educ. 2011;26(3):323-336.
5. Klinger E, Cao X, Douguet A, et al. Annual review of cybertherapy and telemedicine 2009. Designing an ecological and adaptable virtual task in the context of executive functions. Stud Health Technol Inform. 2009;144:248-252.
6. Kortman B, Nicholls K. Assessing for Unilateral Spatial Neglect Using Eye-Tracking Glasses: A Feasibility Study. Occup Ther Health Care. 2016 Oct;30(4):344-355.
7. Nygård L, Starkhammar S, Lilja M. The provision of stove timers to individuals with cognitive impairment. Scand J Occup Ther. 2008;15(1):4-12.
8. Nygård L. The stove timer as a device for older adults with cognitive impairment or dementia: Different professionals' reasoning and actions. Technol Disabil. 2009 11/13;21:53-66.
9. Pinard S, Bottari C, Laliberté C, et al. Design and usability evaluation of COOK, an assistive technology for meal preparation for persons with severe TBI. Disabil Rehabil Assist Technol. 2021 Oct;16(7):687-701.
10. Proffitt R, Sevick M, Chang C-Y, et al. User-centered design of a controller-free game for hand rehabilitation. Games for Health. 2015;4(4):259-264.
11. Starkhammar S, Nygård L. Using a timer device for the stove: experiences of older adults with memory impairment or dementia and their families. Technol Disabil. 2008;20(3):179-191.
12. Zhang L, Abreu BC, Seale GS, et al. A virtual reality environment for evaluation of a daily living skill in brain injury rehabilitation: reliability and validity. Arch Phys Med Rehabil. 2003;84(8):1118-1124.
13. Baum C, Edwards DF. Cognitive performance in senile dementia of the Alzheimer's type: The Kitchen Task Assessment. Am J Occup Ther. 1993;47(5):431-436.
14. Baum CM, Connor LT, Morrison T, et al. Reliability, validity, and clinical utility of the Executive Function Performance Test: A measure of executive function in a sample of people with stroke. Am J Occup Ther. 2008;62(4):446-455.
15. Harridge C, Shah S. A review of meal preparation assessments as a measure of instrumental activities of daily living. N Z J Occup Ther. 1995 1995 Winter;46(1):5-12.
16. Harridge C, Shah S. The Moss Kitchen Assessment Revised. N Z J Occup Ther. 1995 1995 Summer;46(2):5-9.
17. Josman N, Birnboim S. Measuring kitchen performance: what assessment should we choose? Scand J Occup Ther. 2001;8(4):193-202.
18. Porter J, Watson G, Capra S. Food skills assessment tools for people with a mental illness. Aust Occup Ther J. 1998;45(2):65-71.

19. Rocke K, Hays P, Edwards D, et al. Development of a performance assessment of executive function: the Children's Kitchen Task Assessment. Am J Occup Ther. 2008 Sep-Oct;62(5):528-37.
20. Schmelzer L, Stanger H, Hughes R. The Development and Validation of the Planning to Make Meals Performance Measure. OTJR (Thorofare N J). 2022 Apr;42(2):105-114.
21. Neistadt ME. The Rabideau Kitchen Evaluation-Revised: An assessment of meal preparation skill. Occupational Therapy Journal of Research. 1992;12(4):242-255.
