# Supplementary material for: Occupational therapy and cooking: A scoping review and future directions
Source: Scand J Occup Ther. Author manuscript; Available in PMC 2024 Dec 12. (PMC11635739; doi:10.1080/11038128.2023.2267081)
Supplement: Supplemental Table 1 [file NIHMS2038246-supplement-Supplemental_Table_1.pdf]

Supplemental Table 1. Technology and assessment

**Topic**	**Articles**
Focus on technology/virtual reality	[1-12]
Focus on assessment development, validation, or review	[13-21]
