# Supplementary material for: Occupational therapy and cooking: A scoping review and future directions
Source: Scand J Occup Ther. Author manuscript; Available in PMC 2024 Dec 12. (PMC11635739; doi:10.1080/11038128.2023.2267081)
Supplement: Supplemental Table 2 [file NIHMS2038246-supplement-Supplemental_Table_2.pdf]

Supplemental Table 2. Studies included in the review

First Author/ Date	Population/ Country	Context/ Setting	Role of Cooking	Major Findings
Aubin 2009 [56]	Adults with schizophrenia (Canada)	Community dwelling	Evaluated information-processing difficulties experienced during cooking/meal preparation tasks using Perceive, Recall, Plan, and Perform (PRPP) system of task analysis	▪ PRPP results for complex cooking tasks was strongly related to community function measurements.<br>▪ Results support use of PRPP to evaluate functioning in this population.
Aubin 2009 [57]	Adults with schizophrenia (Canada)	Community dwelling	Explored relationships between cooking performance, cognitive functioning, symptoms, and community functioning	▪ Significant associations were found between functional capacity and memory, spatial working memory, planning, and negative symptoms.<br>▪ Planning skills during cooking task were also found to be related to community functioning.
Aubin 2009 [58]	Adults with schizophrenia (Canada)	Community dwelling	Described limitations in information processing skills during cooking and explored whether subgroups have similar functional profiles	▪ Participants in the high-efficiency subgroup were more independent in daily living/community functioning compared to low-efficiency group.
Aubin 2014 [113]	Adults with schizophrenia (Canada)	Community dwelling	Compared information processing skills of people with schizophrenia during cooking tasks with control group of healthy adults	▪ Compared to healthy controls, people with schizophrenia had more errors in information processing during cooking tasks.<br>▪ Accuracy errors may be related to specific skills deficits rather that general processing skills deficits.
Bartlett 2009 [61]	Adults with mental illness (UK)	Acute inpatient setting	Evaluated a group cooking programme based on feedback from group participants and facilitators	▪ Intervention was meeting programme objectives.<br>▪ Evaluation made facilitators more aware of participants' interest in skill development.

Ben-Arye 2016 [59]	Adults undergoing chemotherapy for cancer (Israel)	Outpatient integrated medicine oncology programme	Examined outcomes of integrative medicine cuisine workshop programme, focusing on preparing food to relieve GI systems and fatigue	▪ Workshop participants reported improved quality of life outcomes and a reduction in nutritional and functional concerns.<br>▪ Participants were able to implement recommendations at home.
Berg 2012 [114]	Children (age 8-12) with sickle cell disease (SCD) (USA)	Community dwelling	Compared the executive functioning of children with SCD to matched controls; measures included Children's Kitchen Task Assessment (CKTA)	▪ Children with SCD scored significantly lower than controls on measures of executive functioning, including the CKTA.
Bigelius 2012 [48]	Adults post-stroke (Sweden)	Inpatient unit	Evaluated perceived value and meaning of common cooking task	▪ Selected occupation had high degree of value for this group, with no differences between men and women.<br>▪ VaMO Model was supported.
Bryant 2005 [52]	Adult women with physical or psychosocial impairments (UK)	Community dwelling	Used theory to analyse the cooking environment/ modifications	▪ Use of kitchens as context for occupation is best described through a combination of science and art.<br>▪ Theory can be used as a basis for evidence to support intervention.
Chevignard 2009 [115]	Children (aged 8-14) post-moderate-to-severe brain injury (France)	Inpatient setting and community-dwelling	Compared using naturalistic task (cooking) to standardised assessments for sensitivity to detect executive function deficits	▪ The subjects with brain injuries made a significantly higher number of errors in the cooking tasks than the matched controls.<br>▪ The results indicated that this ecological task was more sensitive than the office-based tests of executive function.
Chevignard 2000 [38]	Adults with dysexecutive syndrome (France)	Community dwelling	Compared performance of executing and generating scripts during daily life activities, including cooking tasks	▪ Subjects demonstrated more errors in script execution than script generation.<br>▪ Script execution may be valid ecological approach to estimating severity of deficits in daily life activities.

Chun 1988 [49]	Adult men with schizophrenia (USA)	Long-term inpatient setting	Examined affective response to group interventions; cooking was one of the three activities provided	▪ Participants rated cooking the highest of the three activities in Osgood's semantic differential (positive affective response).
De Vos 2019 [50]	Adults with mental illness (Canada)	Inpatient setting	Explored participants' experience with group cooking intervention	▪ Three main themes emerged from the interview data: importance of engaging/doing, connections with food, and being involved in a group process<br>▪ Participants valued cooking group and found it important to their recovery.
Dickerson 1997 [27]	Healthy young adults and older adults (USA)	Community dwelling	Compared functional performance of older adults and young adults on cooking versus contrived tasks	▪ Older adults demonstrated age-related declines in motor and process skills regardless of familiarity of tasks.<br>▪ Both groups had higher process performance when give choice of activities.
Dubac 2019 [29]	Adults post-severe brain injury (Canada)	Community dwelling	Examined difficulties experienced with cooking to identify unmet needs	▪ To overcome barriers to cooking, recipes and grocery shopping need to be adapted to meet person's skills/needs.<br>▪ Motivation to cook was linked to mental effort involved in cooking tasks.
Duncombe 2004 [32]	Adults with schizophrenia (USA)	Community dwelling	Compared effectiveness of cooking skills intervention delivered at home versus in a clinic setting	▪ Context had no significant difference on outcomes.<br>▪ Both groups demonstrate improved cooking skills after the intervention.
Eakman 2001 [42]	Adult men post-brain injury (USA)	Rehabilitation centres	Compared hand-on training for preparing food with verbal-only instructions in terms of process recall	▪ Use of hand-on task resulted in better recall.
Eckel 2012 [28]	Older adult women (USA)	Community-dwelling	Identified important cooking activities and barriers to performance	▪ Decline in cooking performance is due both to activity demands and environmental barriers.
Fleming 2020 [43]	Adults post- brain injury (Australia)	Inpatient and community rehabilitation settings	Compared effects of verbal feedback versus experiential feedback on self-awareness during cooking task	▪ Both groups demonstrated improved occupational performance but there was no difference between groups in self-awareness.

Fogel 2020 [117]	Adolescents (age 10-14) with executive function deficits (EFD) (Israel)	Community dwelling	Used Children's Cooking Task (CCT) to compare executive function and multitasking between adolescents with executive function deficits and matched controls	▪ Participants with EFD made significantly more errors, had longer time duration, and had poorer qualitative ratings on CCT compared to controls.<br>▪ The CCT classified a high percentage of the adolescents into their appropriate groups (EFD and control).
Fogel 2022 [116]	Adolescents (age 10-14) with executive function deficits (Israel)	Community dwelling	Used CCT to assess executive function and multitasking abilities before and after a metacognitive, occupation-based programme (FITTED) to address performance in life goals	▪ Significant differences were found in the CCT pre/post assessment, indicating that the adolescents improved their performance after completing the FITTED intervention<br>▪ Actual performance and self-awareness of executive function impairment improved following the intervention, but self-awareness of performance did not.
Foxhall 2014 [47]	Adults post- brain injury (UK)	Inpatient setting	Examined whether evidence-based recommendations for executive function and social skills rehabilitation were incorporated in weekly cooking treatment group	▪ Therapists incorporated executive function training (repetition, errorless learning, meta strategy training) into cooking group but overlooked opportunities for social skill development.
Geusgen 2010 [118]	Healthy adults (Netherlands)	Community dwelling	Examined performance of cooking tasks in familiar versus unfamiliar kitchens	▪ Process skill scores were significantly lower and time to complete task was significantly higher in unfamiliar kitchen.
Grimm 2008 [44]	Adults with schizophrenia (USA)	Long-term inpatient setting	Compared effectiveness of cooking group intervention using a psychoeducation plus an acquisitional approach versus cooking group intervention with solely an acquisitional approach	▪ Level of independence in cooking improved for both groups, but the addition of psychoeducation did not result in greater improvement.

Haley 2004 [53]	Adults with mental illness (UK)	Acute inpatient setting	Explored perspectives of participants of baking group intervention	▪ Participants reported that engaging in baking increased time management skills, sense of purpose, confidence, and skills needed for independent living.<br>▪ A safe and supportive group environment was important to participants.
Hearns 2010 [45]	Healthy adults (university students) (USA)	Community dwelling	Compared hand-on training for preparing food with demonstration-only instructions in terms of process recall	▪ Hand-on learning improved process recall at 15 minutes and 24/48 hours but not for immediate recall.
Helle 2014 [60]	Older adults with mobility limitations (Denmark)	Community dwelling	Examined the validity of accessibility housing standards by noting accessibility problems during common kitchen activities for three groups (rolling walker, wheelchair, and no device)	▪ All three groups experienced accessibility problems, particularly for people using a wheelchair.<br>▪ The housing standards did not support participation in cooking.
Hendry 2016 [119]	Adults post-brain injury (Australia)	Community dwelling	Examined underlying cognitive deficits through standardised cooking assessment in home setting	▪ Errors in cooking tasks result from deficits in a number of cognitive domains.
Hill 2007 [21]	Adult burn survivors (USA)	Inpatient unit	Evaluated therapeutic value of unit cooking group from the perspective of burn survivors	▪ Participants reported that group was a valued treatment activity, addressing socialization, functional activity, and anxiety related to the kitchen setting.
Kondo 1997 [25]	Older adults with motor or visual impairments (USA)	Community dwelling	Evaluated if introduction of microwave and training increased participation in cooking tasks	▪ Having microwave/training increased participation and efficiency in cooking for this group.
Kremer 1984 [51]	Adults with mental illness (USA)	Day treatment programme	Compared affective response to group interventions; cooking was one of three activities provided	▪ Cooking activity scored significantly higher in "evaluation factor" from Osgood's semantic differential (positive feelings) compared to other two activities.
Lock 2012 [46]	Adults with eating disorders (UK)	Inpatient programme and	Evaluated long-term ability and motivation for preparing "normal" meals during and after	▪ Improvement was noted in ability and motivation subscales in participants over

		community dwelling	participation in cooking treatment groups	time, suggesting the interventions supported recovery.
McLean 1987 [23]	Adult with mental illness (USA)	Long-term inpatient setting	Examined outcomes of participation in group cooking skills programme	▪ Attendance in programme was a problem due to institutional factors and therefore it was difficult to evaluate outcomes for the intervention.
Melton 1998 [54]	Adults with mild cognitive impairments (UK)	Community dwelling	Explored experience of cooking with occupational therapist in 1:1 sessions	▪ Participants had powerful individual views about the meaning of cooking.<br>▪ An empowering style of the therapist was found to be important.
Nawate 2008 [39]	Older adults with mild to moderate dementia (Japan)	Community/daycare rehabilitation service	Examined the cognitive and behavioural benefits of group reminiscence intervention using cooking activities	▪ There were significant improvements in scores for cognitive functioning and behaviour after intervention and maintained at 4-week follow-up.
Neistadt 1994 [30]	Adult men post-brain injury (USA)	Inpatient and outpatient programmes	Compared training in cooking and training in parquetry blocks for remediation of fine motor deficits	▪ Both groups showed improvement in measures of coordination but cooking group demonstrated significantly greater improvement in picking up small objects with the dominant hand.
Neistadt 1992 [142]	Adult men post-brain injury (USA)	Inpatient and outpatient programmes	Compared training in cooking and training in parquetry blocks for remediation of constructional skills deficits	▪ Both groups showed task-specific improvements.<br>▪ Functional activities training may be a better way to improve functional performance in this group.
Neistadt 1993 [120]	Adult men post-brain injury (USA)	Long-term rehabilitation programmes	Examined the relationship between cooking and constructional skills	▪ There is an association between cooking performance and constructional skills even when motor skills are controlled for.
Ownsworth 2006 [40]	Adult man post-brain injury (Australia)	Community dwelling	Evaluated neuropsychological and functional outcomes after intensive metacognitive intervention in cooking tasks at home and in volunteer work	▪ After intervention, decreased errors and increased self-correction were noted in each setting.

Ownsworth 2017 [41]	Adults post-brain injury (Australia)	Community dwelling	Compared effectiveness of error-based learning with errorless learning in context of cooking activity at home	▪ Error-based learning was more effective than error-free learning in promoting skill generalization post-intervention.<br>▪ Gains in skills were not sustained over time (at 6-month follow-up).
Poncet 2018 [110]	Adults post-brain injury (France)	Day rehabilitation programme	Evaluated cooking group intervention as part of multidisciplinary day rehabilitation programme	▪ The majority of participants improved in measures of cooking and IADLs at end of programme and maintained these gains (3- and 6-month follow-up).
Poole 2011 [22]	Adults post-stroke (USA)	Community dwelling	Compared cooking performance in subjects after right or left hemisphere stroke to healthy controls, including the associations between cognitive/motor deficits and cooking performance	▪ Both stroke groups took significantly more time to complete the cooking task than the control group.<br>▪ Total errors and level of independence were worse in the left hemisphere stroke group, but individual errors did not significantly differ between the two stroke groups.
Porter 2000 [31]	Adults with mental illness (Australia)	Long-term inpatient setting	Evaluated effectiveness of food skills programme to support transition to the community	▪ Participants demonstrated improvement in food skills indicating the programme may be an effective approach.
Porter 1999 [55]	Adults with mental illness (Australia)	Long-term inpatient and community	Compared food skills, including cooking, of people living in hospital versus those living in the community	▪ People living in the hospital setting had significantly poorer food skills than those living in the community, indicating a need for food skills training to support transition to community living.
Provencher 2012 [121]	Frail older adults (Canada)	Community dwelling	Compared cooking performance (AMPS tasks) in home setting versus clinic setting for subjects with poor and intact executive function	▪ Participants in both groups demonstrated significantly better motor and process skills at home.<br>▪ Participants with poor executive function had notable decrease in process skills in unfamiliar settings, suggesting assessment in home setting may be more accurate.
Provencher 2012 [122]	Frail older adults (Canada)	Community dwelling	Compared cooking performance in home setting versus clinic setting and examined factors which account for difference in performance	▪ Participants showed significantly better performance at home.<br>▪ Better performance at home was associated with lower education, poorer executive

				functions, and higher similarity between settings.
Provencher 2013 [26]	Frail older adults (Canada)	Community dwelling	Compared cooking performance (AMPS tasks) in home setting versus clinic setting	▪ Better performance at home on the process scale was associated with a decrease in some executive functions<br>▪ Better performance in the clinic on the motor scale was mostly related to grip strength.
Schmelzer 2018 [34]	Adults living in poverty (USA)	Community dwelling	Used participatory action research approach to develop and implement programme focusing on managing food resources, including cooking skills	▪ Participants improved on several measures related to managing food resources which supports feasibility and need for the programme.
Schmidt 2015 [37]	Adults post-brain injury (Australia)	Inpatient and community rehabilitation programmes	(Follow-up of Schmidt 2013) Examined maintenance of self-awareness during cooking task in three groups: video plus verbal feedback, verbal feedback, and experiential feedback	▪ Video plus verbal feedback group continued to demonstrate significantly greater improvement in self-awareness compared to the verbal feedback group and the experiential feedback group (at 8-10-week follow-up).
Schmidt 2013 [36]	Adults post-brain injury (Australia)	Inpatient and community rehabilitation programmes	Compared self-awareness during cooking task in three groups: video plus verbal feedback, verbal feedback, and experiential feedback	▪ Video plus verbal feedback was effective in improving self-awareness and did not lead to increased emotional distress.
Tatsumi 2015 [35]	Adults with schizophrenia (Japan)	Inpatient setting	Evaluated effect of cooking group intervention on negative symptoms of schizophrenia	▪ Compared to control group, cooking programme participants demonstrated improved relatedness with therapist and decreased negative symptoms of schizophrenia.

Veneruso 2022 [33]	Adolescents/young adults with autism spectrum disorder (ADS) (Italy)	Community programme	Evaluated cooking intervention targeting improvement of adaptive behaviours, social skills, and severity of ASD-related symptomatology	▪ Participants showed significant improvement in the severity of symptoms and daily living skills.<br>▪ There was no statistically significant improvement in social impairments and adaptive behaviours in the domains of socialization and communication.
Wang 2019 [112]	Adults post-brain injury (USA)	Community dwelling	Compared feasibility of automatic prompting system versus user-controlled tablet during cooking	▪ Automatic prompting showed decreased need for assistance and was rated as easier and less stressful for participants but user-controller system offered more flexibility in timing.
Whiteman 1989 [24]	Adults with physical disabilities (UK)	Community dwelling	Examined value of using microwaves for cooking for people with physical disabilities	▪ Microwaves supported cooking engagement though they were not a complete replacement for conventional cooking appliances.
Yaddaden 2020 [123]	Occupational therapists (Canada)	NA	Explored therapists' views on the utility of assistive technology (COOK) for supporting cooking for older adults with mild cognitive impairment and Alzheimer's disease	▪ According to OTs, depending on their diagnosis, older adults have different functional profiles and need different interventions during cooking.<br>▪ COOK has greater potential to be implemented with older adults with mild cognitive impairment than those with Alzheimer's disease.
Zarshenas 2021 [111]	Adult woman post-severe stroke (Canada)	Supported community residence	Used case study to pilot use of assistive technology (COOK) to support cooking performance	▪ COOK significantly increased independence by decreasing the required external assistance and the task performance errors while increasing appropriate responses to safety issues during meal preparation.<br>▪ The improvements were maintained at 6-week follow-up.
